# Enzymatic synthesis of calcium lactobionate from cheese whey permeate as a value-added ingredient

**DOI:** 10.3168/jdsc.2025-0834

**Published:** 2025-10-18

**Authors:** Usman Amin, Chi Kong Yeung, Haotian Zheng

**Affiliations:** 1 Southeast Dairy Foods Research Center, Department of Food, Bioprocessing and Nutrition Sciences, North Carolina State University, Raleigh, NC 27695; 2 Animal Science Department, California Polytechnic State University, San Luis Obispo, CA 93407

## Abstract

Lactobionic acid (LBA) and its salt form (e.g., lactobionate [LBN]) have emerged as high-value-added functional ingredients in food and pharmaceutical applications, such as acidulant, antioxidant, metal chelator, and carrier in drug delivery systems. Enzymatic oxidation has been employed as a nontoxic, cost-effective, and environmentally friendly approach for the synthesis of LBA/LBN. The current study investigated, first, the feasibility of producing calcium lactobionate (Ca-LBN) via enzymatic oxidation using cheese whey permeate as a substrate at high concentration, and second, the bioactivity of the resulting Ca-LBN. The production experiment was performed using reconstituted cheese whey permeate solution (300 g·L^−1^ lactose) as a substrate, Ca(OH)_2_ as a titrant base, oxidase (dosage: 400 U·kg^−1^ lactose), and catalase (dosage: 168,000 U·kg^−1^ lactose) in a laboratory bioreactor. Target critical control parameters, such as pH 6.40; dissolved oxygen: 44%; and temperature: 38°C, were defined and monitored using an industrial human-machine interface (HMI) to ensure operational stability. The consumption of Ca(OH)_2_ was used to calculate real-time molar conversion rate (MCR_RT_) and accumulative molar conversion yield (MCY) according to the pH-stat method. Enzymatic oxidation reaction continued for 7 h, and MCY was observed at nearly 99%. The MCR_RT_ rapidly reached a plateau value of ∼470 mmol·h^−1^ within 20 min of the process. The critical operational parameters remained controlled by the HMI cascade, suggesting that the process is scalable. The DPPH-radical scavenging and ferrous ion chelating activity of the obtained LBN could not be confirmed based on the colorimetric assays used in the present work; however, characterization processes need to be further optimized. The obtained knowledge may be applied to the scalable production of LBA/LBN, enabling higher yields and an efficient manufacturing process.

## Highlights

• Enzymatic synthesis of Ca-LBN at high substrate concentration was studied.
• The enzymatic oxidation process lasted 7 hours and achieved an MCY of ~99%.
• Process parameters (e.g., temperature, pH, and DO%) were monitored and maintained.
• Ca-LBN exhibited potential 2,2-diphenyl-1-picrylhydrazyl-radical scavenging and metal chelation activities.
• The knowledge obtained may be used for scale-up production of Ca-LBN.

## Graphical Abstract

**Figure:**
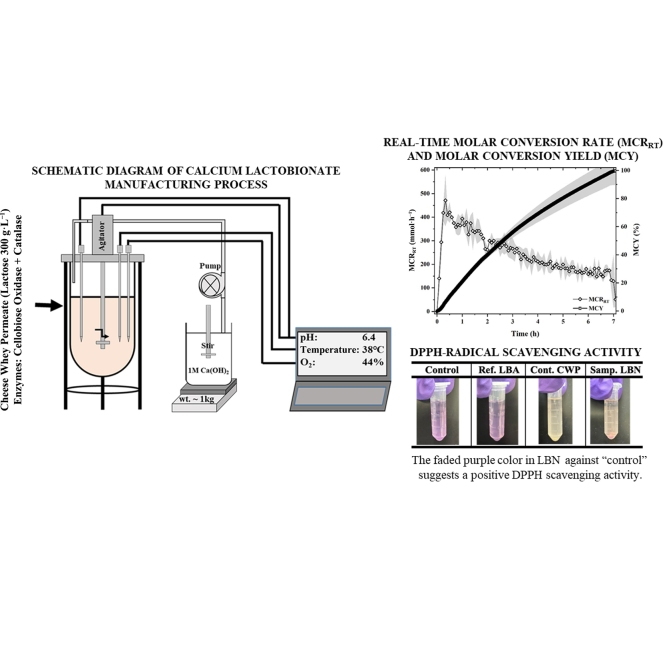
**Summary:** In this study, calcium lactobionate (Ca-LBN) was manufactured through an enzymatic oxidation process using cheese whey permeate as a substrate and Ca(OH)_2_ as a neutralizing base. The process was operated based on the pH-stat method using a benchtop bioreactor. The operational performance of the enzymatic conversion process was analyzed by estimating the real-time molar conversion rate (MCR_RT_) and molar conversion yield (MCY). The enzymatic oxidation process lasted for nearly 7 hours and achieved an MCY of nearly 99%. The critical operational parameters, such as dissolved oxygen (DO%), temperature, and pH, were monitored and controlled during the manufacturing process. The manufactured Ca-LBN showed possible 2,2-diphenyl-1-picrylhydrazyl-radical scavenging and ferrous ion chelating activities; future work is needed to establish feasible and reliable methods for quantifying their bioactivities. This work suggests that employed processing parameters were effective in achieving a high LBN yield and may be adopted by the manufacturing industry for scalable production of LBN.

Lactobionic acid (**LBA**; molecular weight: 358.30 Da; pKa: 3.8), also known as 4-O-β-D-galactopyranosyl-D-gluconic acid, is an aldonic acid comprises of a galactose moiety linked to a gluconic acid molecule via an ether-like linkage (Gutiérrez et al., 2012; National Center for Biotechnology Information, 2025). In general, LBA has been recognized as one of the higher value-added organic acid ingredients as compared with the conventional ones, such as lactic acid, citric acid, acetic acid, and so on; however, the production of LBA is relatively lower than the conventional organic acids (Alonso et al., 2013). Lactobionic acid is a natural polyhydroxy acid having numerous hydroxyl groups, making it very hygroscopic, soluble in water, and forms a gel with about 14% moisture content (Gutiérrez et al., 2012; Cardoso et al., 2019). Commercially, LBA and its salt forms, such as sodium (Na)-, calcium (Ca)-, and magnesium (Mg)-lactobionates (**LBN**), have been widely used for pharmaceutical, medical, and food applications due to their excellent metal chelating, antioxidant, and emulsifying properties. For pharmaceutical applications, LBA and LBN can be used for antiaging (Green et al., 2009), skincare (Tasic-Kostov et al., 2012), calcium supplementation (Frutos et al., 1997), solubility enhancer for antibiotics (e.g., amoxicillin; Haseena et al., 2021), and so on. Regarding medical applications, LBA or LBN is used as one of the crucial ingredients in the University of Wisconsin (UW) solution, which is used as a preservation solution in organ transplantation (Southard et al., 1990). In cosmetics, LBA is used as a humectant and antioxidant in skincare products, such as moisturizers and antiaging formulations (Algiert-Zielińska et al., 2019). For food applications, Ca-LBN has been approved by the US Food and Drug Administration (**FDA**) to be used as a food additive as a firming agent in dry pudding mixes at an amount not greater than that required to produce the intended effect (FDA, 2018). Despite the benefits and applications mentioned, heavy metal chelating and antioxidant properties as potential value propositions of LBA and LBN still need to be explored further for creating or extending their economic value.

The LBA/LBN can be produced from lactose by different techniques, including electro-catalytic oxidation (Kokoh and Alonso-Vante, 2006), bacterial (Miyamoto et al., 2000), and enzymatic (Nordkvist et al., 2007) oxidation. Energy-intensive processes and heavy metal residuals in the final LBN products make the chemical oxidation methods environmentally unfriendly, and they are unfavored for food and cosmetic applications. Biochemical methods, such as enzymatic processing methods, are, in general, considered lower energy cost techniques and safe for human consumption compared with commercial chemical processes (Hossain et al., 2025). The mechanism of enzymatic conversion process involves oxidation of lactose while continuously reducing the pH of the system. To maintain optimal enzyme activity, a sodium or calcium base solution is added to the reaction system for maintaining the process pH. This method is referred to as the pH-stat method and has been investigated in previous works (Hua et al., 2007; Nordkvist et al., 2007). Owing to the cation (e.g., Na^+^ or Ca^2+^) introduced in the reaction system, the yield product is the cation-based LBN.

Despite the previously mentioned advantages of enzymatic conversion methods, empirical research on enzymatic production of LBA and LBN from permeate streams derived from cheese whey and skim milk is limited in the literature. The lack of knowledge regarding key processing parameters for LBN manufacturing from dairy permeates hindered industrial commercialization of LBN as a value-added dairy ingredient. Nordkvist et al. (2007) conducted a processing study on enzymatic synthesis of Na-LBN utilizing carbohydrate oxidase for lactose oxidation. In this referred work, lactose solution was used as a substrate. The authors reported that the conversion yield was dependent on the various operation parameters (e.g., temperature, oxygen level, substrate concentration, use of catalase, and so on). In another scale-up study, Hua et al. (2007) reported 98% conversion of lactose to lactobionic acid, validating the efficacy of the enzymatic conversion method. It is important to note that the authors used whey permeate as a substrate and investigated the enzymatic reaction rate as a function of dissolved oxygen tension. However, the substrate concentration used in the referred work was relatively low, which was 50 g·L^−1^. This would result in a low production rate of LBA and Na-LBN. To the best of our knowledge, the processing feasibility of enzymatic production of LBN from cheese whey permeate (**CWP**) with high substrate content is not available in the literature.

Given the high lactose content (∼80%) of CWP and its relatively low economic value, it is motivated to process CWP into high-value-added ingredients, such as LBA and LBN. Therefore, the objectives of the current study were to (1) investigate the processing feasibility of enzymatic manufacturing of LBN using CWP solution containing high lactose content as a substrate and (2) investigate the bioactivities of the manufactured LBN.

The CWP was kindly provided by Hilmar Ingredients (Hilmar, CA). The enzymes, including cellobiose oxidase (LactoYIELD) and catalase (Catazyme), were provided by Novonesis North America (formerly known as Chr. Hansen, Franklinton, NC). Calcium hydroxide [Ca(OH)_2_] was purchased from Fisher Scientific (Waltham, MA).

Scalable Ca-LBN production experiments were conducted using an automated benchtop scale bioreactor (RALF Basic bioreactor, Bioengineering AG, Switzerland) controlled by an industrial human-machine interface (**HMI**) system. The reaction substrate was made of 2 L of CWP solution in deionized (**DI**) water containing 300 g·L^−1^ lactose with a pH adjusted to pH 6.4. The substrate solution in the glass vessel of the RALF bioreactor was maintained at 38°C by a heating jacket as a part of the bioreactor system. The bioreactor setting for dissolved oxygen (**DO%**) level was set as 44%; the DO% was dynamically adjusted by a control valve connected to an O_2_ tank, and the actual DO% in the reaction system was monitored using a DO% sensor mounted on the bioreactor. The system pressure was monitored throughout the reaction, and it was within a range of 0.3 to 4.0 kPa for all the performed experiments. Enzymes, including oxidase and catalase, were added to the substrate solution at the starting point with dosage rates of 400 U·kg^−1^ lactose and 168,000 U·kg^−1^ lactose, respectively. According to Nordkvist et al. (2007), it has been assumed that oxidase (*E_ox_*) follows a ping-pong reaction mechanism for the bioconversion of lactose as substrate (*S*) into the anion of LBA as product (*P*) as follows:

[1] Eox+S→←to.5exk-1

[2] $$E_{red}\;+\;O_{2}\;\overset{k_{3}}{\to }\;E_{ox}\;+\;H_{2}O_{2}.$$

Here, *E_ox_* and *E_red_* express the oxidized and the reduced forms of the oxidase enzyme (*E*), respectively, whereas *k_i_* refers to the different rate constants for substrate binding and substrate-product conversion during the enzymatic reaction process. Catalase was used to break down H_2_O_2_ produced during the oxidation process (Equation 2) for the proper functioning of oxidase.

About 1,000 g of 1 *M* Ca(OH)_2_ solution was loaded into the base reservoir to maintain the designated pH 6.4 according to the pH-stat method. The base reservoir was placed on a weight balance connected to an HMI system for monitoring the base mass change, which is used for computing the molar amount of LBN (Mol_LBN_*_−t_*) produced at a certain time point (*t*) according to the following equation:

[3] $$\begin{array}{c} \mathrm{Mo}l_{\mathrm{LBN}-t}=V_{t}\;\times M\times 2, \end{array}$$

where *V_t_* and *M* are the consumed volume of base solution at a time point (*t*) in liters, and its molarity (1 *M*), respectively. *V_t_* was calculated based on the measured mass change and density of the base solution, Ca(OH)_2_. This LBN quantification method, based on the consumption of base solution and the original molar amount of lactose (Mol_LAC_), is adopted from a previous work with reasonable modification (Nordkvist et al., 2007). The real-time molar conversion rate (**MCR_RT_**, mmol·h^−1^) and the accumulative molar conversion yield (**MCY**, %) were estimated according to the following equations:

[4] $$\begin{array}{c} \mathrm{MC}R_{\mathrm{RT}}=\frac{\mathrm{Mo}l_{\mathrm{LBN}-t2}-\;\mathrm{Mo}l_{\mathrm{LBN}-t1}}{t2-t1}, \end{array}$$

[5] $$\begin{array}{c} \mathrm{MCY}\;\left(\%\right)=\frac{\mathrm{Mo}l_{\mathrm{LBN}-t}}{\mathrm{Mo}l_{\mathrm{LAC}}}\times 100\%. \end{array}$$

The referred LBN quantification method was validated by quantification of the produced H_2_O_2_ as a co-product from the enzymatic substrate oxidation reaction, according to Nordkvist et al. (2007), where separate manufacturing experiments were conducted without the addition of catalase. The validation experiment was performed for a period of 4 h. Aliquots of sample mixture were extracted from the reaction vessel at 1-h intervals throughout the experiment. The sample aliquots were diluted 6,250-fold from their original concentration. Then, 3 mL of the diluted sample was mixed with 75 μL of ABTS solution (28 g·L^−1^; 2,2′-azino-bis(3ethylbenzothiazoline-6-sulfonic acid) and 15 μL of horseradish peroxidase (500 U·mL^−1^). The absorbance of the mixture was measured immediately at 419 nm using a UV-visible spectrophotometer (UV-1800, Shimadzu, Kyoto, Japan). A standard curve of H_2_O_2_ (0–40 μ*M*) was used to calculate the H_2_O_2_ produced at different time periods, as described in the literature (Nordkvist et al., 2007, 2003).

The liquid LBN products were characterized for 2,2-diphenyl-1-picrylhydrazyl (**DPPH**) radical scavenging activity and Fe-based metal chelation activity (**MCA**); additionally, DI water, commercial chemical grade LBA sourced from ThermoFisher Scientific (Waltham, MA), and CWP solution were used as the blank control, reference, and control, respectively, for comparison with the manufactured Ca-LBN. The LBA and CWP were reconstituted in DI water at 300 m*M* concentration. The obtained LBN dispersions were also adjusted to a concentration of 300 m*M*. Then, all the prepared sample dispersions were treated by means of microfiltration followed by centrifugation for removing colloidal particles. The samples were either filtered by 0.45-µm or 0.1-µm syringe filters (Millex, MilliporeSigma, Burlington, MA); the filtrates were then centrifuged at 5,000 × *g* for 5 min at room temperature (Sorvall Legend M 21R, ThermoFisher Scientific, Waltham, MA). To measure the DPPH-radical scavenging capacity, 2.9 mL of DPPH solution (0.1 m*M* in ethanol [80%, vol/vol]) was mixed with 0.1 mL of each testing sample according to Cardoso et al. (2019). A method control sample was prepared by replacing the testing sample with DI water (2.9 mL of DPPH + 0.1 mL of DI water). The mixtures were incubated in the dark for 30 min, and the absorbance was determined at 517 nm. For MCA, 0.4 mL of each sample was mixed with 0.2 mL of 1 m*M* FeSO_4_ and 0.4 mL of 5 m*M* ferrozine. The mixture was diluted with DI water to make the final volume of 4 mL. The mixtures were incubated for 10 min at room temperature, and the absorbance was measured at 562 nm (Cardoso et al., 2019). Both DPPH-radical scavenging activity and MCA were measured using the following equation:

[6] $$\begin{array}{c} \mathrm{DPPH}\;\mathrm{or}\;\mathrm{MCA}\;\left(\%\right)=1-\frac{Abs_{sample}}{Abs_{control}}\times 100, \end{array}$$

where *Abs_sample_* and *Abs_control_* are the absorbance of the sample and reagent control, respectively.

All LBN production and characterization experiments were performed no less than in duplicate, and the results are expressed as mean ± SD.

The key in-process parameters, pH and DO%, are shown in Figure 1. The bioreactor was able to maintain the reaction within a narrow pH range of 6.3 to 6.5 (Figure 1a), indicating the effectiveness of cascade controller of the bioreactor. It took almost 1 h for the DO% level to reach the designated level, and the DO% level fluctuated between 30% and 60% afterward until nearly the end of the reaction (Figure 1b). At the end of the reaction period, the DO% was raised to close to 100%, indicating a low usage of DO%, and therefore the completion of the oxidation process (Figure 1b).

**Figure 1:**
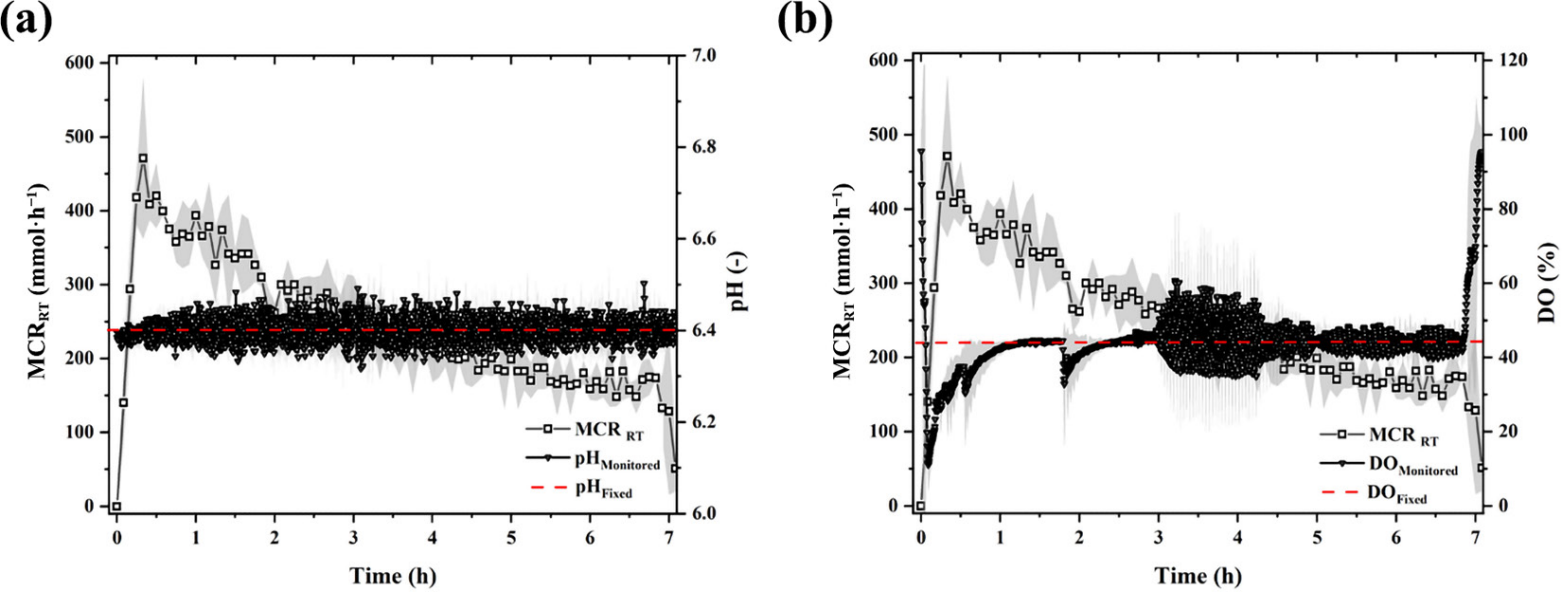
(a) Real-time molar conversion rate (MCR_RT_) versus variation in pH and (b) MCR_RT_ versus variation in dissolved oxygen (DO%) over the Ca-LBN production cycle performed for 7 h. The shaded regions indicate the standard deviation.

Initially, MCR_RT_ linearly increased and reached the plateau value at ∼470 mmol·h^−1^ within 20 min of the reaction (Figure 1a). This is because a large amount of substrate (lactose) was available for the enzymatic oxidation. Passing the peak rate, MCR_RT_ continuously decreased throughout the reaction period (7 h) until the full LBN conversion was reached. The MCR_RT_ decreasing curve fluctuated as shown in Figure 1; this is likely due to the fluctuation of DO% during the reaction course. Hua et al. (2007) showed that the LBA enzymatic reaction rate was positively related to the DO% following a linear relationship. Chidar et al. (2022) also reported that the DO% determined both LBA conversion yield and conversion rate (also known as productivity, g·L^−1^·h^−1^). The current results and the referred work suggest that DO% was a critical processing factor.

In contrast, the real-time MCY (%) continuously increased throughout the reaction course for 7 h until nearly 99% of lactose was converted to the LBA anion (Figure 2a). The decrease in MCR_RT_ in the period of 1 to 7 h seemed to decrease the slope of MCY curve accordingly. It is important to note that the peak MCR_RT_ was at ∼470 mmol·h^−1^. This rate is more than 10 times higher than the reported “base addition rate” in a previous study of enzymatic manufacturing of LBA (Nordkvist et al., 2007). The “base addition rate” is equivalent to MCR_RT_ as used in the present work. Such a significant reaction rate difference may be attributed to the greatly higher substrate concentration (e.g., 300 g·L^−1^) used in the present work as compared with the referred work (e.g., 50 g·L^−1^). Moreover, it seemed that the high MCR_RT_ was responsible for the effective lactose conversion. Figure 2a showed that nearly 100% lactose was converted to the anion of LBA in about 7 h. Nordkvist et al. (2007) showed that the yield of LBA reached the plateau at around 8 h. These conversion yields are higher compared with ∼70% as found in another study, in which the substrate concentration was 100 g·L^−1^ (Chidar et al., 2022). The lower conversion yield might be attributed to the difference in DO% between the referred and the present works. Chidar et al. (2022) did not report the DO% level in the LBA conversion process.

**Figure 2:**
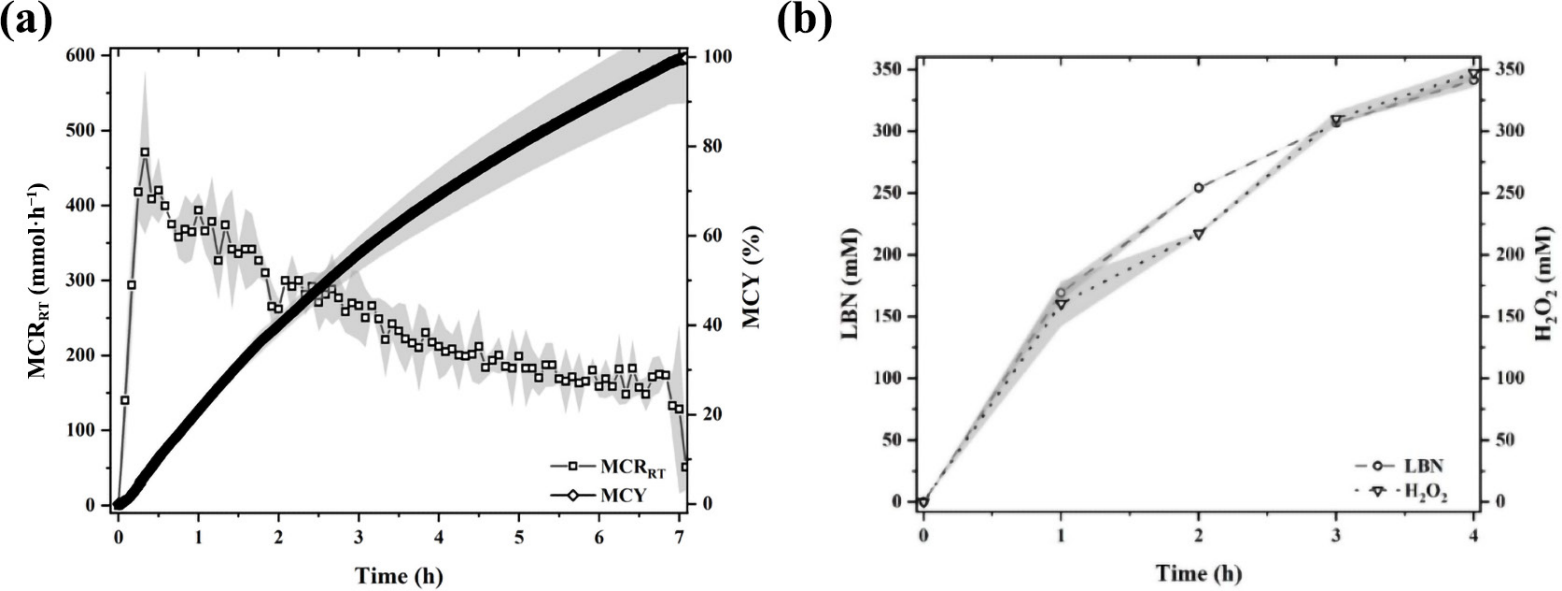
(a) Real-time molar conversion rate (MCR_RT_) and molar conversion yield (MCY) of enzymatically produced Ca-LBN at an oxidase/lactose ratio of 400 U·kg^−1^. (b) The validation experiment was performed without the addition of catalase to confirm the pH-stat-based concentration of LBN with the amount of H_2_O_2_ for real-time monitoring of the process. The shaded regions refer to the standard deviation.

In addition to the pH-stat quantification method to estimate the molar amount of synthesized anion of LBA based on Equation 3, an additional quantification method was performed for validation purposes. The validation was done by performing a separate LBN production experiment without the addition of catalase, the enzyme that catalyzes the breakdown of H_2_O_2_. According to Equations 1 and 2, the molar amount of H_2_O_2_ generated as a co-product during the enzymatic synthesis of LBA is equivalent to the molar amount of the anion of LBA produced. Therefore, the validation experiment is based on the quantification of H_2_O_2_ produced; the molar amount of H_2_O_2_ was used to indicate the amount of anion of LBA produced. Figure 2b shows that, over the reaction period, the LBN amount obtained from the consumed base solution generally agreed with the measured amount of H_2_O_2_. The nearly matched curves, as shown in Figure 2b, suggest that the MCR_RT_ and MCY quantifications based on the consumed base solution were reliable and indicative of the true production dynamics of the anion of LBN.

Free radical scavenging activity and metal chelating activity were measured using DPPH-radical scavenging and ferrous ion chelation assays, respectively, and results are presented in Figure 3. The observed color-fading effects in sample-reagent mixtures may be interpreted as positive activities of DPPH scavenging and metal chelation. Regarding the DPPH-radical scavenging capacity analysis, it is necessary to emphasize that the LBA, LBN, and CWP samples were pretreated by micro-filtration and centrifugation to remove colloidal particles. The colloidal particles presented in the tested samples [e.g., residual Ca(OH)_2_ in LBN] may possibly induce light scattering, and therefore, interfere with spectrophotometry absorbance measurements. Indeed, the removal of unknown colloidal particles can be beneficial for absorbance measurements; however, it is reasonable to argue that the elimination of colloidal particles could cause underestimation of the actual or the absolute DPPH scavenging activity and metal chelating property of the original bulk samples due to the fact that the removed particles might possess the referred bioactivities. Therefore, to prevent misinterpretation of quantitative bioactivities of LBA, CWP, and LBN, the quantified values of DPPH scavenging (%) and MCA (%) based on measured absorbances are not shown, and only “qualitative” results are presented in this work. For instance, images of individual samples are shown in Figure 3 to demonstrate the color-fading effects (e.g., LBN against water control), suggesting the positive DPPH scavenging and MCA effects for the LBN sample.

**Figure 3:**
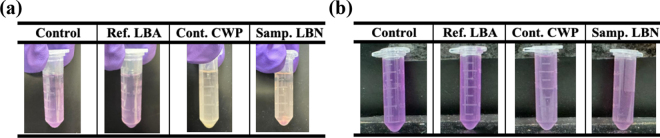
Visualization of color comparison among samples from DPPH-radical scavenging activity characterization (a) and metal chelation activity evaluation (b). Control = DI water as a blank control; Ref. LBA = LBA solution as the reference sample; Cont. CWP = CWP solution as a control sample for comparing with the testing sample, Ca-LBN; Samp. LBN = diluted LBN solution used as the testing sample.

The ferrous and ferric ions chelating activities for the anion of LBA/LBN had been reported in the literature (Isaacson et al., 1989; Cardoso et al., 2019). Ferrous and ferric ions may interact with hydrogen peroxide (H_2_O_2_) and lipid peroxides, respectively, producing free hydroxyl radicals and lipid peroxyl radicals (Algiert-Zielińska et al., 2019). Therefore, the ferrous and ferric ions chelating properties may induce secondary antioxidant activities; this makes LBA/LBN suitable for applications in cosmetology for eliminating the generation of free radicals. It is interesting to observe a color-fading effect in the CWP sample (Figure 3). This observation suggested that CWP and LBN both had DPPH-radical scavenging activity. These phenomena may be attributed to the presence of bioactive compounds in the CWP solution. The CWP contains bioactive peptides, organic acids, such as lactic acid, and sialyloligosaccharides (Beucler et al., 2005; Barile et al., 2009). Additionally, the application of intense heat treatments during drying can instigate the Maillard reaction, leading to the formation of Maillard reaction products (Morales et al., 1996; Tanguy et al., 2017). These compounds could contribute to the antioxidant and metal chelation properties of CWP. Indeed, a recent study showed that CWP had a significant DPPH-radical scavenging activity, and such activity was even enhanced after heat treatment (e.g., sterilization treatment, 110°C for 6 min; Lamothe et al., 2025). Future research efforts are needed to optimize the colorimetric assays for eliminating the negative impacts from the colloidal particles.

In conclusion, the present study demonstrated a feasible enzymatic processing method for the manufacturing of Ca-LBN from a reconstituted CWP system containing high substrate concentration (i.e., 300 g·L^−1^ lactose). The process was carried out using an automated benchtop bioreactor controlled by an HMI system, which is integrated with a series of cascades mimicking industrial-scale bioreactors. The key operational parameters, including temperature, pressure, pH, base consumption, and DO%, were either controlled or monitored; therefore, the reported process is fully scalable to industrial operation. The reported process resulted in a high molar conversion yield of approximately 99% within 7 h. The obtained Ca-LBN showed possible activities in scavenging DPPH free radicals and chelating ferrous ions. The characterization methods for quantifying antioxidant and metal chelating activities need further optimization and investigation. Overall, the obtained knowledge of the work provides the industry with operational parameters for obtaining a high yield of the anion of LBA/LBN within a relatively shorter processing time. Future research efforts may be focused on optimizing drying technology to obtain dried LBN ingredients for food and cosmetic applications.
